# Analysis of Synovial Fluid Metabolic Profile in Patients with Knee Osteoarthritis Using Spectroscopic Magnetic Resonance Metabolomics

**DOI:** 10.1055/s-0044-1790217

**Published:** 2024-12-21

**Authors:** Paulo Ricardo Picon Alves, Diego Pinheiro Aguiar, Aline Cordeiro Fernandes Ladeira, Gilson Costa dos Santos, Eduardo Branco de Sousa

**Affiliations:** 1 Divisão de Ensino e Pesquisa, Instituto Nacional de Traumatologia e Ortopedia Jamil Haddad, Rio de Janeiro, RJ, Brasil; 2 Departamento de Farmácia, Faculdade de Ciências Biológicas e Saúde, Universidade do Estado do Rio de Janeiro, Rio de Janeiro, RJ, Brasil; 3 Departamento de Genética, Instituto de Biologia Roberto Alcantara Gomes, Universidade do Estado do Rio de Janeiro, Rio de Janeiro, RJ, Brasil; 4 Laboratório de Metabolômica, Instituto de Biologia Roberto Alcantara Gomes, Universidade do Estado do Rio de Janeiro, Rio de Janeiro, RJ, Brasil; 5 Departamento de Cirurgia Geral e Especializada, Faculdade de Medicina, Universidade Federal Fluminense, Rio de Janeiro, RJ, Brasil

**Keywords:** magnetic resonance spectroscopy, metabolomics, osteoarthritis, synovial fluid

## Abstract

**Objective** The present study aimed to evaluate the metabolic profile of synovial fluid in patients with knee osteoarthritis (KOA) and its correlation with clinical data.

**Materials and Methods** We collected synovial fluid samples from the knees of 50 subjects with KOA undergoing total knee arthroplasty from October 2019 to December 2020. For each patient, we evaluated the clinical data from the medical record, the radiographic osteoarthritis grade, and the preoperative fasting blood glucose levels. The samples underwent metabolomic analysis by 1H magnetic resonance spectroscopy, and we compared the spectra using multivariate and univariate analyses.

**Results** Most patients were female (66%). The subjects had an average age of 67.96 ± 7.08 years old and an average body mass index (BMI) of 32.51 ± 5.25 kg/m ^2^ . Clinical and metabolic evaluations revealed that 88% of patients were hypertensive and presented higher levels of valine, arginine, and citrate than non-hypertensive subjects.

**Conclusion** Metabolomic analysis of synovial fluid cannot classify osteoarthritis patients per their clinical characteristics.

## Introduction

Knee osteoarthritis (KOA) is characterized by articular cartilage destruction, synovial inflammation, and subchondral bone thickening. 1 This condition affects the joint as an organ. 2 Metabolic syndrome is a set of clinical and metabolic disorders that increase the mortality risk and present with obesity, arterial hypertension, dyslipidemia, glucose intolerance, and a proinflammatory state. 3 Some research tried subdividing osteoarthritis (OA) into phenotypes, 4 5 including OA associated with metabolic syndrome. 4 5 6

A promising tool for this phenotypical subclassification is metabolomics, 7 8 which aims to understand cellular biological interactions by identifying the molecules within a biological sample. 9 Metabolomics is distinct for evaluating the resultant of genetic and epigenetic phenomena interacting with the environment. 10 One of the most used analytical platforms is magnetic resonance spectroscopy, which has the following advantages: reproducibility, not requiring prior preparation, and sample indestructibility during analysis, favoring the performance of numerous examinations with the same material. 11 12 Thus, synovial fluid metabolomics has been identified as a potential tool for determining OA-related biomarkers. 11 13

The present study aimed to analyze the metabolic profile of synovial fluid in patients with KOA and correlate the findings with clinical data.

## Materials and Methods

### Patient Selection and Clinical Data Collection

The current study included 50 patients with KOA undergoing total knee arthroplasty (TKA) from October 2019 to December 2020. All patients signed the informed consent form, and the Research Ethics Committee approved this study under CAAE number 23695019.5.0000.5273.

The study included patients with KOA according to clinical-radiographic diagnostic criteria. 14 We excluded patients with an active infection in the operated limb, those who underwent previous surgery on the same knee, those with autoimmune diseases or genetic syndromes, those with a positive serological test for hepatitis B, C, and HIV, or any combination of these viruses, with malignant neoplasms or under antineoplastic chemotherapy, subjects treated with immunosuppressants or glucocorticoids, and patients with a collected volume of synovial fluid lower than 1 mL, which is deemed insufficient for analysis.

We analyzed the metabolic profile of the synovial fluid of patients with KOA and compared the findings with clinical, radiographic, and laboratory profiles. To investigate the influence of aging on the metabolic behavior of KOA, we divided patients between those up to and over 70 years old. We arbitrarily chose 70 years as a cutoff, as this age is considered a good parameter for the proposed surgery. 15 Due to the close relationship between obesity and KOA, we tested the metabolic profile of patients according to their body mass indices (BMIs).

We assessed the participants' medical records to collect the following information, summarized in Table 1 :

**Table 1:** Demographic data of patients included in the study

Variable	Osteoarthritis (n = 50)
**Gender**	
Male	17 (34%)
Female	33 (66%)
**Age (years)**	67.96 ± 7.08
**Weight (kg)**	84.07 ± 14.91
**Height (m)**	1.61 ± 0.1
** Body mass index (kg/m ^2^ ) **	32.51 ± 5.25
**Obesity**	
Normal	4 (8%)
Overweight	12 (24%)
Obese	34 (68%)
**Osteoarthritis severity**	
Ahlbӓck modified by Keyes et al. 17 III	3 (6%)
Ahlbӓck modified by Keyes et al. 17 IV	7 (14%)
Ahlbӓck modified by Keyes et al. 17 V	40 (80%)
**Side**	
** Right**	22 (44%)
** Left**	28 (56%)
**Arterial hypertension**	
Yes	44 (88%)
No	6 (12%)
**Diabetes mellitus**	
Yes	11 (22%)
No	39 (78%)
**Dyslipidemia**	
Yes	4 (8%)
No	46 (92%)
**Heart disease**	
Yes	3 (6%)
No	47 (94%)

**Notes:** Absolute data on age, weight, height, and body mass index are presented as mean and standard deviation values. Other data are presented as absolute and relative frequencies.

1) Gender, age, and laterality of the operated knee;
2) Anthropometric data: weight in kilograms (kg) and height in meters (m) for BMI calculation in kg/m ^2^ . Each participant was classified as normal (BMI ranging from 18.5–24.9), overweight (BMI ranging from 25-29.9), or obese (BMI ≥ 30); 16
3) Clinical aspects (collected from the anesthetic evaluation form): presence or absence of systemic arterial hypertension, type 2 diabetes mellitus, dyslipidemia, and heart disease;
4) Fasting blood glucose levels: Preoperative fasting blood glucose level in milligrams per deciliter (mg/dL).

We evaluated and classified the preoperative radiographs per the Ahlbäck system modified by Keyes et al. 17 Since the sample consisted only of patients undergoing TKA, we divided the patients into three groups (grades III, IV, and V).

We tabulated the data collected from the medical records in a Microsoft Excel spreadsheet (Microsoft Corp., Redmond, WA, USA) and used the GraphPad Prism for Windows 9.0 software (GraphPad Software, La Jolla, CA, USA) for statistical analysis. We presented data from age, weight, height, and BMI as mean ± standard deviation (SD), and data regarding arterial hypertension, dyslipidemia, heart disease, diabetes, and kidney disease as absolute and relative frequencies.

### Metabolic Analysis of Synovial Fluid by Magnetic Resonance Spectroscopy

The surgeon collected synovial fluid samples under sterile conditions, during TKA, before opening the joint capsule, using needle aspiration. After collection, the samples were placed in a thermal bag at 4 C, transported to the research laboratory, transferred to microtubes, identified, centrifuged at 2,000 x g for 5 minutes, and decanted. The supernatants, containing cell-free synovial fluid, were frozen at -80 C, without dilution.

**Fig. 1 Schematic of sample processing.**
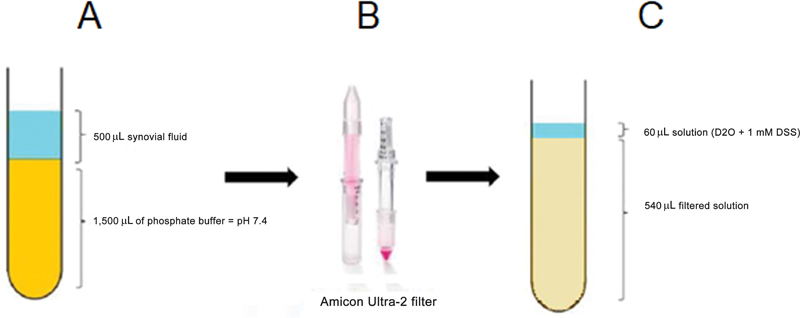
Schematic illustration of mixing the synovial fluid with phosphate buffer. Next, the photo of the Amicon filter, taken from the manufacturer's website ( https://www.merckmillipore.com/BR/pt/product/Amicon-Ultra-2-Centrifugal-Filter-Unit,MM_NF-UFC200324 ) on June 13, 2021. Finally, the addition of 60 μL of a solution containing the standard substance (2,2-dimethyl-silapentane-2-5-sulfonate [DSS]) to 540 μL of the filtered solution, totaling 600 μL.

Two hours before metabolomic analysis, we thawed the samples at room temperature and centrifuged them at 7,168 x g for 20 minutes. Next, we mixed 500 μL of the supernatant with 1,500 μL of phosphate buffer, at 50 mM and pH 7.4 ( Fig. 1A ). To eliminate interference from large molecules, we filtered mixtures of synovial fluid with phosphate buffer using Amicon Ultra-2 membranes (MERCK-Millipore, Burlington, MA, USA, Fig. 1B ), with a 3-kDa mesh (#UFC200324) and centrifuged the samples at 4,032 x g for 20 minutes. After centrifugation, we transferred 540 μL of the filtered solution to a new microtube and added 60 μL of a solution containing 1 mM 2,2-dimethyl-silapentane-2-5-sulfonate (DSS) and deuterated water (D _2_ O). ( Fig. 1C ). To verify the effect of other compounds in the buffer and filter, we prepared a control sample, which contained phosphate buffer instead of the synovial fluid. We subsequently removed the compounds detected in this sample from the analysis.

**Fig. 2 Schematic with photographs of the equipment used.**
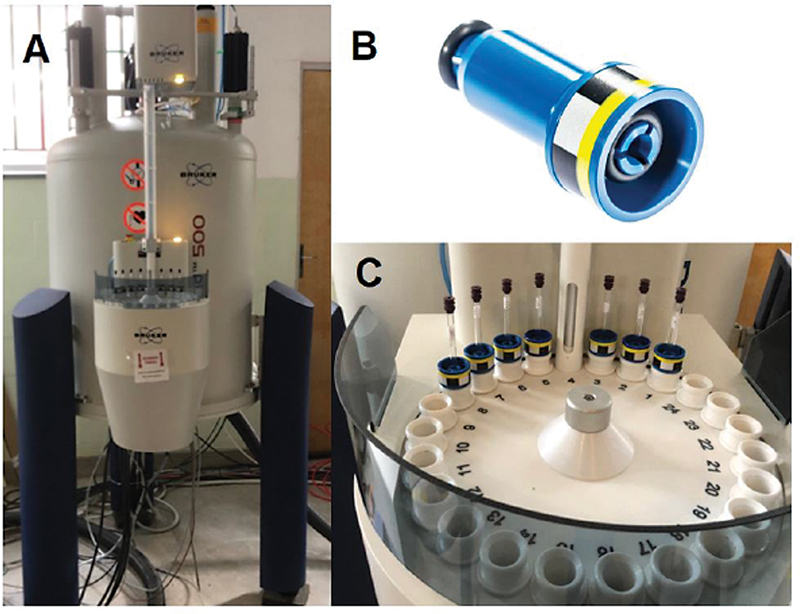
( **A** ) Image of the Bruker Avance III HD 500 MHz device for metabolomics. ( **B** ) The photograph of the sample spinner used in this research from taken from https://cortecnet.com/5-mm-pom-spinner-500-mhz.html on June 13, 2021. ( **C** ) Visualization of the device sampler during preparation for analysis. Note that the samples have already been inserted into the first eight positions. Each sample, contained in a 5-mm tube, is attached to the rotor for insertion into the sampler positions.

We placed the duly identified microtubes containing the mixtures in a thermal bag at 4 C for transportation to the metabolomics laboratory. There, we manually transferred 600 μL from the Eppendorf tubes (Eppendorf, Hamburg, Germany) to the 5-mm diameter magnetic resonance tubes. Samples analysis occurred at the Bruker Avance III HD spectrometer (Bruker Biospin, Ettlingen, Germany), Fig. 2A ), operating at 500 MHz and a controlled probe temperature of 300 K. We coupled the 5-mm magnetic resonance spectroscopy tubes to a device called a sample spinner *,* Fig. 2B ) and inserted them into the 24 holders of the device's sampler for analysis ( Fig. 2C ). The study of the metabolic profile of the synovial fluid samples followed the description from Sousa et al. 13

## Results

**Fig. 3 Division of patients according to the OA severity, age, and gender.**
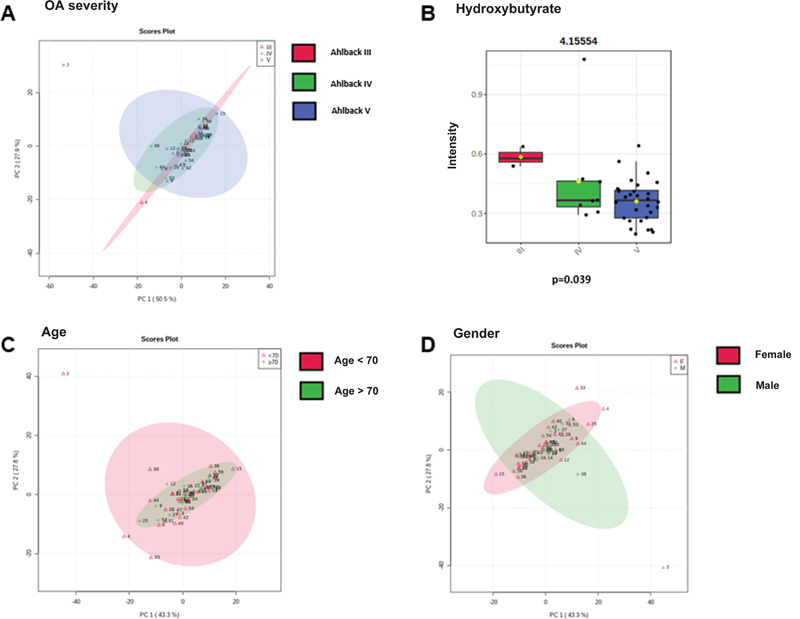
( **A** ) Principal component analysis (PCA) graphs from the three groups: grades III (red diagram), IV (green diagram), and V (blue diagram) according to the Ahlbäck classification. The score plot of PC 2 versus PC 1, in which each point corresponds to a patient. ( **B** ) Boxplot demonstrating differences in the concentration of the metabolite 3-hydroxybutyrate (4.15554) between groups. ( **C** ) Principal component analysis graphs from the groups of subjects under 70 years old (red diagram) and over 70 years old (green diagram). The score plot of PC 2 versus PC 1, in which each point corresponds to a patient. ( **D** ) Principal component analysis graphs from the female (red diagram) and male (green diagram) groups. The score plot of PC 2 versus PC 1, where each point corresponds to a patient. Note: * *p* -value obtained by analyzing multiple t-tests in Graph Pad Prism 9.0.

The principal component analysis (PCA) was unable to classify subjects according to Ahlbäck grades III, IV, and V ( Fig. 3A ), age subgroups ( Fig. 3C ), or gender subgroups ( Fig. 3D ) per the metabolic profile. In turn, the univariate analysis found differences in the concentration of the metabolite 3-hydroxybutyrate ( *p* = 0.039; Fig. 3B ) between groups according to KOA severity. Statistical analysis with multiple t-tests did not reveal significant differences between metabolites in age or gender subgroups ( *p* > 0.05).

**Fig. 4 Division of patients according to body mass index (BMI).**
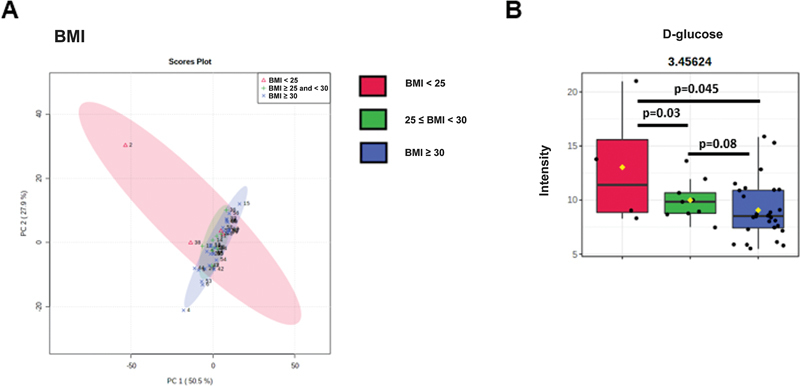
( **A** ) Principal component analysis (PCA) graphs from the three groups: normal BMI (red diagram), overweight (green diagram), and obesity (blue diagram). ( **B** ) The score plot of PC 2 versus PC 1, where each point corresponds to a patient. ( **C** ) Boxplot demonstrating differences in the concentration of the metabolite D-glucose (3.45624) between the groups. Note: * *p* -value obtained by analyzing multiple t-tests in Graph Pad Prism 9.0.

This analysis could not present a robust separation of subjects per BMI and metabolic profile ( Fig. 4A,B ). Statistical analysis with multiple t-tests revealed no differences between groups ( *p* > 0.05). The univariate analysis found differences in the concentration of the metabolite D-glucose ( *p* = 0.037; Fig. 4C ) between groups.

**Fig. 5 Division of patients according to fasting blood glucose levels, diabetes mellitus, dyslipidemia, and heart disease.**
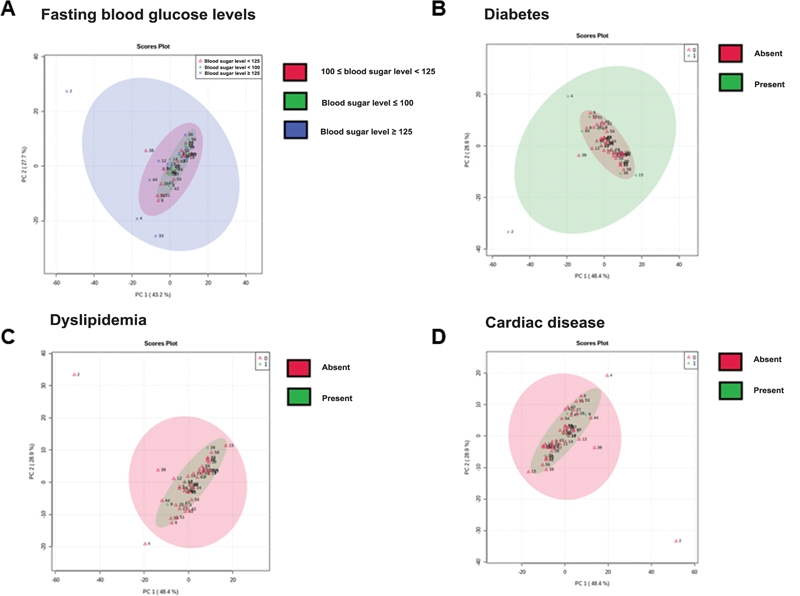
( **A** ) Principal component analysis (PCA) graphs from the three groups: normal fasting blood sugar levels (green diagram), prediabetes (red diagram), and diabetes (blue diagram) per the metabolic profile. ( **B** ) PCA graphs from the two groups: with (green diagram) or without (red diagram) diabetes mellitus. ( **C** ) PCA graph from the two groups: with (green diagram) or without (red diagram) dyslipidemia. ( **D** ) PCA graph from the two groups: with (green diagram) or without (red diagram) heart disease. The score plot of PC 2 versus PC 1, where each point corresponds to a patient.

**Fig. 6 Discrimination of patients according to the diagnosis of arterial hypertension.**
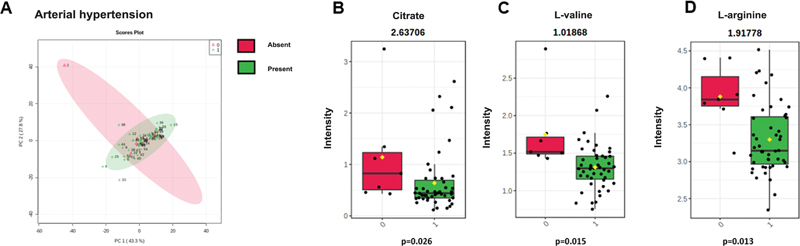
( **A** ) Principal component analysis (PCA) graphs from the two groups: with (green diagram) or without (red diagram) arterial hypertension. The score plot of PC 2 versus PC 1, where each point corresponds to a patient. Boxplots demonstrating the difference in the concentration of the metabolites ( **B** ) citrate (2.63706), ( **C** ) L-valine (1.01868), and ( **D** ) L-arginine (1.91778) between groups. Note: * *p* -value obtained by analyzing multiple t-tests in Graph Pad Prism 9.0.

Moreover, PCA was unable to solidly segment subjects per fasting blood glucose levels ( Fig. 5A ), diabetes mellitus ( Fig. 5B ), dyslipidemia ( Fig. 5C ), heart disease ( Fig. 5D ), and hypertension ( Fig. 6A ) according to the metabolic profile. Statistical analysis with multiple t-tests did not reveal differences between groups according to fasting blood glucose levels, diabetes, dyslipidemia, or heart disease ( *p* > 0.05). However, the univariate analysis found differences in the concentration of the metabolites L-valine ( *p* = 0.015; Fig. 6B ), L-arginine ( *p* = 0.013; Fig. 6C ), and citrate ( *p* = 0.026; Fig. 6D ).

## Discussion

The main findings of our study were that the metabolite 3-hydroxybutyrate showed a variation inversely proportional to OA severity; meanwhile, the metabolites 3-hydroxybutyrate and L-valine showed decreasing levels depending on the radiographic KOA severity. We also found lower values of arginine, valine, and citrate in the subgroup of hypertensive patients, and lower blood glucose levels in obese patients.

Recently, a systematic review of the literature concluded that OA results in a wide variety of metabolic pathway alterations, with no consensus regarding a metabolite or panel for potential diagnostic use. 18

The univariate analysis showed that the 3-hydroxybutyrate metabolite varied inversely proportional to OA severity. Another metabolite with a similar behavior was L-valine, with decreasing levels concerning OA degree but no statistical significance. The ratio between serum valine and histidine levels has been proposed as a diagnostic marker for OA. 19 It is inferred that increased serum level of branched-chain amino acids is involved with the increased synthesis of inflammatory cytokines in OA, 7 but this was not well established in synovial fluid. Another study of synovial fluid using mass spectrometry analysis identified increased levels of 28 metabolites in the advanced KOA group; these molecules resulted from the lipid, glycolipid, and Krebs cycle metabolism, suggesting an aberrant metabolic activity associated with mitochondrial dysfunction and autophagy. 20 Wang et al. 21 reported an increased concentration of the metabolite N-α-acetyl-L-asparagine in association with severe KOA, indicating that this molecule level may predict the severity of the disease. However, the analysis was targeted, unlike our study, which did not identify this metabolite despite the larger number of patients.

It was not possible to discriminate participants according to the two age groups or genders using PCA, probably because all patients had severe KOA. Welhaven et al. 22 analyzed the synovial fluid of patients with meniscal, anterior cruciate ligament, or both injuries, concluding that men and women had different metabolite profiles, and suggested that these data could be further used to evaluate the development of posttraumatic OA. On the other hand, Zhai et al. 23 suggested that phenylalanine could be a plasma marker for the progression of bilateral radiographic KOA in women. In our study, analyses occurred only on the synovial fluid from patients with primary KOA, potentially explaining the difference between these findings.

The multivariate analysis demonstrated no differences in synovial fluid metabolism according to BMI. This result surprised us, as significant differences in serum metabolism between obese and non-obese patients with KOA have already been demonstrated in blood samples. 24 As a systemic disease, the metabolite profile in the blood of obese and non-obese subjects may not mirror the synovial environment. Another plausible explanation for our result is the influence of the subclassification we used, stratified into three groups, instead of a comparison between obese and non-obese people. Although the multivariate analysis found no difference, the synovial glucose concentration was lower in the obese group. Although the glucose metabolite is not directly identified as a BMI-related biomarker, we know that increased metabolite levels, such as mannose, are linked to overweight and obesity. 25 We speculate that this occurs in the joints of obese subjects because of the higher glucose consumption by synovial cells.

Comparisons of the metabolic profile of the synovial fluid of patients with and without diabetes and per fasting blood glucose levels did not reveal differences in multivariate and univariate analyses. The relationship between diabetes and OA has been extensively researched, and the existence of a diabetes-induced phenotype has been suggested. 26 One factor potentially influencing our results is the non-consideration of hypoglycemic drugs or insulin, which directly interferes with glucose metabolism. Furthermore, the classification as diabetic or non-diabetic relied on self-declaration during the interview instead of laboratory diagnostic methods.

Hypertension apparently contributes to OA pathogenesis through biochemical and biomechanical aspects by interfering with joint microcirculation. 27 Multivariate analysis could not distinguish groups of patients with or without hypertension per their metabolic profile. However, valine, arginine, and citrate levels were higher in subjects without the disease. This result suggests citrate consumption favors inflammatory pathways in the joints of these patients, corroborating the hypothesis of a greater inflammatory influence in OA associated with this chronic disease. 27 Valine also demonstrated lower synovial concentrations in hypertensive patients. The increase in the serum leucine/histidine ratio can also serve as a biomarker for KOA diagnosis as it relates to collagen degradation. 19 Arginine was another metabolite in lower levels in the hypertension group. Zhang et al. 28 pointed to a reduced serum arginine level as a potential biomarker of advanced OA, inferring that this diminution originates from a compensatory increase in collagen synthesis in severe OA. Other research demonstrated increased serum arginine levels in patients with severe OA undergoing total knee and hip arthroplasty compared with healthy subjects. 29 pointed to a reduced serum arginine level as a potential biomarker of advanced OA, inferring that this diminution originates from a compensatory increase in collagen synthesis in severe OA. Other research demonstrated increased serum arginine levels in patients with severe OA undergoing total knee and hip arthroplasty compared with healthy subjects. 27 The synovial drainage disturbance, resulting from the vascular impairment caused by hypertension, may explain the lower synovial concentration of some metabolites in patients with OA and hypertension. Werdyani et al. 30 demonstrated, after a metabolomic analysis of plasma from patients with hip OA and KOA, the existence of three endotypes of the disease: muscle weakness, arginine deficit, and low-grade inflammation. Although these authors analyzed plasma, such findings corroborate our data since hypertensive patients had lower arginine levels.

We found no differences when evaluating the metabolic profile of the synovial fluid of patients with or without heart disease. However, the diagnosis of heart disease was based on preanesthetic evaluation, and only 6% of the samples were from subjects with these conditions.

One of the strengths of our study was the number of synovial fluid samples from KOA patients. Another positive point of our research was the lower variability in the joint disease grade, comparing patients only with advanced stages of joint destruction.

A main limitation of our study was the classification of some clinical conditions based on data from the standard preanesthetic assessment form by self-declaration. Another limitation was the analysis of only synovial fluid, limiting the comparison with other plasma-using studies.

## Conclusion

Synovial fluid multivariate analyses did not reveal differences between KOA patients according to clinical characteristics. The metabolites 3-hydroxybutyrate and L-valine showed lower levels depending on radiographic severity; obese patients had lower blood glucose levels; and hypertensive patients presented lower arginine, valine, and citrate levels.
