# Supplementary material for: Analysis of Synovial Fluid Metabolic Profile in Patients with Knee Osteoarthritis Using Spectroscopic Magnetic Resonance Metabolomics
Source: Rev Bras Ortop (Sao Paulo). 2024 Dec 21;59(6):e958–65. doi: 10.1055/s-0044-1790217 (PMC11663051; doi:10.1055/s-0044-1790217)
Supplement: Related texts

# Research article: Análise do perfil metabólico do líquido sinovial de pacientes com osteoartrite do joelho por meio da metabolômica por ressonância magnética espectroscópica

**Keywords:** espectroscopia de ressonância magnética, líquido sinovial, metabolômica, osteoartrite

## Abstract

**Objetivo** Avaliar o perfil metabólico do líquido sinovial de pacientes com osteoartrite do joelho (OAJ) correlacionando com os dados clínicos.

**Métodos** Coletamos amostras de líquido sinovial do joelho de 50 indivíduos com OAJ durante artroplastia total do joelho entre outubro de 2019 e dezembro de 2020. Avaliamos, para cada paciente, os dados clínicos do prontuário médico, o grau de artrose radiográfica e a glicemia de jejum pré-operatória. As amostras foram submetidas à análise metabolômica por ressonância magnética (RM) 1H, e os espectros foram comparados por meio de análises multivariadas e univariadas.

**Resultados** O sexo feminino prevaleceu (66%), a média de idade foi 67,96 ± 7,08 anos e a média do índice de massa corporal (IMC) foi de 32,51 ± 5,25 kg/m2. Nas avaliações clínicas e metabólicas, 88% dos pacientes eram hipertensos, e encontramos maiores níveis de valina, arginina e citrato em comparação com pacientes não hipertensos.

**Conclusão** A análise metabolômica do líquido sinovial não conseguiu classificar os pacientes com OA de acordo com as características clínicas.

## Introdução

A osteoartrite do joelho (OAJ) é uma doença caracterizada pela destruição da cartilagem articular, inflamação sinovial e espessamento do osso subcondral, 1 podendo ser entendida como uma doença que acomete a articulação como um órgão. 2 A síndrome metabólica é um conjunto de perturbações clínicas e metabólicas que aumentam o risco de mortalidade, participando desse contexto a obesidade, a hipertensão arterial, a dislipidemia, a intolerância à glicose e um estado pró-inflamatório. 3 Algumas pesquisas têm procurado subdividir a osteoartrite em fenótipos, 4 5 incluindo a osteoartrite associada à síndrome metabólica. 4 5 6

Uma das ferramentas promissoras utilizadas para essa subclassificação em fenótipos é a metabolômica, 7 8 que visa compreender as interações biológicas celulares mediante a identificação das moléculas presentes em uma amostra biológica, 9 sendo distinta por avaliar a resultante dos fenômenos genéticos e epigenéticos interagindo com as ações do ambiente. 10 Uma das plataformas analíticas mais empregadas é a espectroscopia por ressonância magnética (RM), que apresenta como vantagens a reprodutibilidade, a independência de preparo prévio e a indestrutibilidade da amostra durante a análise, o que favorece a realização de inúmeras análises com o mesmo material. 11 12 Assim, a metabolômica do líquido sinovial tem sido apontada como ferramenta potencial para a identificação de biomarcadores relacionados à osteoartrite. 11 13

O objetivo desse estudo foi analisar o perfil metabólico do líquido sinovial de pacientes com osteoartrite, correlacionando os achados com dados clínicos.

## Materiais e Métodos

## Seleção dos pacientes e coleta dos dados clínicos

Foram incluídos neste estudo 50 pacientes com OAJ submetidos à artroplastia total do joelho (ATJ), no período de outubro de 2019 a dezembro de 2020. Todos os pacientes assinaram o Termo de Consentimento Livre e Esclarecido, e a pesquisa foi aprovada pelo Comitê de Ética em Pesquisa (CAAE: 23695019.5.0000.5273).

Foram incluídos no estudo pacientes com diagnóstico de OAJ conforme critérios diagnósticos clínico-radiográficos. 14 Foram excluídos pacientes com infecção ativa no membro operado; pacientes submetidos à cirurgia prévia no mesmo joelho; pacientes com doenças autoimunes, ou com síndromes genéticas; pacientes com teste sorológico positivo para hepatite B, C e/ou HIV; pacientes com neoplasias malignas ou em uso de quimioterápicos antineoplásicos; pacientes em uso de imunossupressores ou de glicocorticoides; e pacientes com volume de líquido sinovial coletado inferior a 1 mL, considerado insuficiente para as análises.

Neste estudo, o perfil metabólico do líquido sinovial de pacientes com OAJ foi analisado e comparado com o perfil clínico, radiográfico e laboratorial. No intuito de investigar a influência do envelhecimento no comportamento metabólico da OAJ, dividimos os pacientes entre os com idade até 70 e aqueles acima de 70 anos. Escolhemos arbitrariamente 70 anos como corte, pois esta idade é apontada como um bom parâmetro etário para a cirurgia proposta. 15 Em função da relação íntima entre obesidade e OAJ, testamos o perfil metabólico dos pacientes de acordo com o índice de massa corporal (IMC).

Foram analisados os prontuários dos participantes para a coleta das seguintes informações, resumidas na Tabela 1 :

**Tabela 1:** Dados demográficos dos pacientes incluídos no estudo

Variável	Osteoartrite (n = 50)
**Sexo**	
Masculino	17 (34%)
Feminino	33 (66%)
**Idade (anos)**	67,96 ± 7,08
**Peso (kg)**	84,07 ± 14,91
**Altura (m)**	1,61 ± 0,1
** Índice de massa corporal (kg/m ^2^ ) **	32,51 ± 5,25
**Obesidade**	
Normal	4 (8%)
Sobrepeso	12 (24%)
Obeso	34 (68%)
**Gravidade da osteoartrite**	
Ahlbӓck modificada por Keyes et al. 17 III	3 (6%)
Ahlbӓck modificada por Keyes et al. 17 IV	7 (14%)
Ahlbӓck modificada por Keyes et al. 17 V	40 (80%)
**Lado**	
Direito	22 (44%)
Esquerdo	28 (56%)
**Hipertensão arterial**	
Sim	44 (88%)
Não	6 (12%)
**Diabetes mellitus**	
Sim	11 (22%)
Não	39 (78%)
**Dislipidemia**	
Sim	4 (8%)
Não	46 (92%)
**Cardiopatia**	
Sim	3 (6%)
Não	47 (94%)

**Notas:** Os dados absolutos de idade, peso, altura e índice de massa corporal foram expressos por valores de média e desvio padrão. Os demais dados foram expressos por valores de frequência absoluta e frequência relativa.

1) Sexo, idade e lateralidade do joelho operado;
2) Dados antropométricos: peso, expresso em quilogramas (kg), e altura, em metros (m), para cálculo do IMC, em kg/m ^2^ . Cada participante foi classificado como normal (IMC entre 18,5–24,9), sobrepeso (IMC entre 25–29,9) ou obeso (IMC ≥ 30); 16
3) Aspectos clínicos, coletados a partir da ficha de avaliação anestésica: presença ou ausência de hipertensão arterial sistêmica, diabetes mellitus tipo 2, dislipidemia e doença cardíaca;
4) Glicemia de jejum: Resultado do nível sérico, pré-operatório, da glicemia de jejum, expresso em mg/dL (miligramas por decilitro).

As radiografias pré-operatórias dos joelhos operados foram avaliadas e classificadas, conforme a classificação de Ahlbäck modificada por Keyes et al. 17 Uma vez que a amostra foi composta apenas por pacientes submetidos à ATJ, dividimos os pacientes em três grupos (grau III, IV e V).

Os dados coletados dos prontuários foram tabulados em planilha Microsoft Excel (Microsoft Corp., Redmond, WA, EUA). Para a análise estatística foi utilizando o software GraphPad Prism for Windows 9.0 (GraphPad Software, La Jolla, CA, EUA). Os dados correspondentes à idade, ao peso, à altura e ao IMC foram apresentados sob a forma de média ± desvio padrão. Já os dados referentes à presença de hipertensão arterial, de dislipidemia, de cardiopatia, de diabetes e de doença renal foram apresentados em frequência absoluta e em frequência relativa.

## Análise metabólica do líquido sinovial por RM

As amostras de líquido sinovial foram coletadas pelo cirurgião, de forma estéril, durante o procedimento de ATJ, antes da abertura da cápsula articular, por punção aspirativa. Após a coleta, as amostras foram acondicionadas em bolsa térmica a 4° C, transportadas até o laboratório de pesquisa, transferidas para microtubos, identificadas, centrifugadas a 2.000 x g durante 5 minutos, e decantadas. Os sobrenadantes, contendo líquido sinovial livre de células, foram congelados a -80° C, sem qualquer diluição.

Duas horas antes da análise metabolômica, as amostras foram descongeladas em temperatura ambiente e centrifugadas a 7.168 x g, por 20 minutos. A seguir, 500 μL do sobrenadante foram misturados com 1.500 μL do tampão fosfato, a 50 mM, em pH 7,4 ( Fig. 1A ). Para eliminar a interferência de moléculas grandes, as misturas de líquido sinovial com tampão fosfato foram filtradas em membranas Amicon Ultra-2 (MERK-Millipore, Burlington, MA, EUA, Fig. 1B ), com malha de 3 kDa (#UFC200324), e centrifugadas a 4.032 x g, por 20 minutos. Após a centrifugação, 540 μL da solução filtrada foram transferidas para um novo microtubo e foram adicionados 60 μL de uma solução, contendo 1 mM de 2,2-dimetil-silapentano-2-5-sulfonato (DSS) e água deuterada (D _2_ O) ( Fig. 1C ). Para verificar o efeito de outros compostos presentes no tampão e no filtro, foi preparada uma amostra controle, na qual o líquido sinovial foi trocado por tampão fosfato. Os compostos detectados nesta amostra foram posteriormente retirados da análise.

**Fig. 1 Esquema do processamento das amostras.** Ilustração esquemática da mistura do líquido sinovial com o tampão fosfato *.* Na sequência, a foto do filtro Amicon, retirada do site do fabricante ( https://www.merckmillipore.com/BR/pt/product/Amicon-Ultra-2-Centrifugal-Filter-Unit,MM_NF-UFC200324 ), no dia 13/06/2021. Por fim, 60 μL de uma solução contendo a substância padrão (DSS) é adicionada à 540 μL da solução filtrada, totalizando 600 μL.

Os microtubos, contendo as misturas e devidamente identificados, foram acondicionados em bolsa térmica a 4°C, para serem transportados até o laboratório de metabolômica, no qual foram transferidos manualmente os 600 μL dos tubos Eppendorf (Eppendorf, Hamburgo, Alemanha) para os tubos de RM, de 5 mm de diâmetro. As amostras foram analisadas com o espectrômetro Bruker Avance II HD (Bruker Biospin, Ettlingen, Alemanha, Fig. 2A ), que operou a 500 MHz, com a temperatura da sonda controlada a 300 K. Os tubos de RM de 5 mm foram acoplados a um dispositivo chamado rotor da amostra ( *sample spinner* , Fig. 2B ) e inseridos nas 24 posições ( *holders* ) do amostrador do aparelho para as análises ( Fig. 2C ). O perfil metabólico das amostras de líquido sinovial foi analisado conforme descrito em Sousa et al. 13

**Fig. 2 Esquema com fotografias dos equipamentos utilizados.** ( **A** ) Imagem do aparelho Bruker Avance III HD 500 MHz utilizado para metabolômica. ( **B** ) Fotografia do mesmo rotor ( *sample spinner* ) utilizado nesta pesquisa. A fotografia foi coletada do site https://cortecnet.com/5-mm-pom-spinner-500-mhz.html , no dia 13/06/2021. ( **C** ) Visualização do amostrador do aparelho, durante preparação para as análises. Observe que as amostras já foram inseridas nas oito primeiras posições. Cada amostra, contida em um tubo de 5 mm, é acoplada ao rotor, para ser inserida nas posições do amostrador.

## Resultados

A análise por análise do componente principal (ACP) não foi capaz de classificar os indivíduos de acordo com os graus III, IV e V de Ahlbäck ( Fig. 3A ), subgrupos por idade ( Fig. 3C ), nem de acordo com sexo ( Fig. 3D ), conforme o perfil metabólico. Por sua vez, a análise univariada encontrou diferenças na concentração do metabólito 3-hidroxibutirato ( *p* = 0,039; Fig. 3B ) entre os grupos de acordo com a gravidade da OAJ. A análise estatística com múltiplos testes t não evidenciou diferenças significativas entre os metabólitos nos grupos por faixa etária e sexo ( *p* > 0,05).

**Fig. 3 Discriminação dos pacientes de acordo com a gravidade da osteoartrite, com a idade e com o sexo.** ( **A** ) Gráfico da análise pelo PCA entre os três grupos: graus III (diagrama vermelho), IV (diagrama verde) e V (diagrama azul) conforme a classificação de Ahlbäck. O *Score-Plot* , de PC 2 *versus* PC 1, em que cada ponto corresponde a um paciente. ( **B** ) *Box-plot* demonstrando diferenças na concentração do metabólito 3-hidroxibutirato (4.15554) entre os grupos. ( **C** ) Gráfico da análise pelo PCA entre os dois grupos, abaixo de 70 anos (diagrama vermelho) e acima de 70 anos (diagrama verde). O *score-Plot* , de PC 2 *versus* PC 1, em que cada ponto corresponde a um paciente. ( **D** ) Gráfico da análise pelo PCA entre os dois grupos, sexo feminino (diagrama vermelho) e masculino (diagrama verde) pelo perfil metabólico. *Score-Plot* , de PC 2 *versus* PC 1, onde cada ponto corresponde a um paciente. Nota: *valor de *p* obtido pela análise de múltiplos testes t no Graph Pad Prism 9.0.

Esta análise não foi capaz de apresentar uma separação robusta nos indivíduos de acordo com o IMC conforme o perfil metabólico ( Fig. 4A,B ). A análise estatística com múltiplos testes t não evidenciou diferenças entre os grupos ( *p* > 0,05). Já a análise univariada encontrou diferenças na concentração do metabólito D-glicose ( *p* = 0,037; Fig. 4C ) entre os grupos.

**Fig. 4 Discriminação dos pacientes de acordo com o índice de massa corporal.** ( **A** ) Gráfico da análise pelo PCA entre os três grupos: IMC normal (diagrama vermelho), sobrepeso (diagrama verde) e obeso (diagrama azul). ( **B** ) *Score-Plot* , de PC 2 *versus* PC 1, em que cada ponto corresponde a um paciente. e ( **C** ) *Box-plot* demonstrando diferenças na concentração do metabólito D-glicose (3.45624) entre os grupos. Abreviatura: IMC, índice de massa corporal. Nota: *valor de *p* obtido pela análise de múltiplos testes t no Graph Pad Prism 9.0.

Também não foi capaz de apresentar uma segmentação sólida entre os indivíduos, de acordo com a medida da glicemia de jejum ( Fig. 5A ), nem de acordo com o diagnóstico de diabetes mellitus ( Fig. 5B ), dislipidemia ( Fig. 5C ), cardiopatia ( Fig. 5D ) e hipertensão ( Fig. 6A ), conforme o perfil metabólico. A análise estatística com múltiplos testes t não evidenciou diferenças entre os grupos de acordo com a medida da glicemia de jejum, diagnóstico de diabetes, dislipidemia ou cardiopatia ( *p* > 0,05). Contudo, a análise univariada encontrou diferenças na concentração dos metabólitos L-valina ( *p* = 0,015; Fig. 6B ), L-arginina ( *p* = 0,013; Fig. 6C ) e citrato ( *p* = 0,026; Fig. 6D ).

**Fig. 5 Discriminação dos pacientes de acordo com a glicemia de jejum, diabetes mellitus, dislipidemia e doença cardíaca.** ( **A** ) Gráfico da análise pelo PCA entre os três grupos: glicemia de jejum normal (diagrama verde), pré-diabetes (diagrama vermelho) e diabetes (diagrama azul) conforme o perfil metabólico. ( **B** ) Gráfico da análise pelo PCA entre os dois grupos: presença (diagrama verde) ou ausência do diagnóstico de diabetes mellitus (diagrama vermelho). ( **C** ) Gráfico da análise pelo PCA entre os dois grupos: presença (diagrama verde) ou ausência do diagnóstico de dislipidemia (diagrama vermelho). ( **D** ) Gráfico da análise pelo PCA entre os dois grupos: presença (diagrama verde) ou ausência do diagnóstico de cardiopatia (diagrama vermelho). *Score-Plot* , de PC 2 *versus* PC 1, em que cada ponto corresponde a um paciente.

**Fig. 6 Discriminação dos pacientes pelo diagnóstico de hipertensão arterial.** ( **A** ) Gráfico da análise pelo PCA entre os dois grupos: presença (diagrama verde) ou a ausência do diagnóstico de hipertensão arterial (diagrama vermelho). *Score-Plot* , de PC 2 *versus* PC 1, em que cada ponto corresponde a um paciente. *Box-plot* demonstrando diferença na concentração do metabólito ( **B** ) citrato (2.63706), ( **C** ) L-valina (1.01868) e ( **D** ) L-arginina entre os grupos (1.91778). Nota: *valor de *p* obtido pela análise de múltiplos testes t no Graph Pad Prism 9.0.

## Discussão

Os principais achados de nosso estudo foram que o metabólito 3-hidroxibutirato apresentou variação inversamente proporcional à gravidade da OA, enquanto os metabólitos 3-hidroxibutirato e L-valina apresentaram níveis decrescentes em função da gravidade radiográfica da OAJ. Também encontramos menores valores de arginina, valina e citrato no subgrupo de pacientes hipertensos, e a glicose apresentou menor concentração nos obesos.

Recentemente, uma revisão sistemática da literatura concluiu que há uma grande variedade de vias metabólicas alteradas na osteoartrite, não havendo consenso a respeito de um metabólito ou painel que pudesse ser utilizado para o diagnóstico. 18

A análise univariada evidenciou que o metabólito 3-hidroxibutirato apresentava variação inversamente proporcional à gravidade da OA. Outro metabólito que apresentou comportamento similar foi a L-valina, com níveis decrescentes em relação ao grau de OA, mas que não foi estatisticamente significativo. A razão entre os níveis séricos de valina e da histidina já foi proposta como marcador diagnóstico da OA. 19 Infere-se que a elevação no nível sérico dos aminoácidos de cadeia ramificada esteja envolvida com o aumento da síntese de citocinas inflamatórias na OA, 7 o que ainda não foi bem estabelecido no líquido sinovial. Outro estudo analisou mediante análise por espectrometria de massa o líquido sinovial, identificando 28 metabólitos aumentados no grupo OAJ avançada, relacionados ao metabolismo lipídico, glicolipídico e do ciclo de Krebs, sugerindo uma atividade metabólica aberrante associada à disfunção mitocondrial e a autofagia. 20 Wang et al. 21 identificaram que o aumento na concentração do metabólito N-α-acetil-L-asparagina estava associado com a OAJ grave, podendo ser indicado para predizer a gravidade da doença. Entretanto, a análise realizada foi do tipo direcionada, diferentemente da análise realizada no presente estudo, que não identificou o referido metabólito, embora tenha analisado um número maior de pacientes.

Não foi possível discriminar os participantes pelos dois grupos etários, tampouco de acordo com o sexo, pela ACP, provavelmente pelo fato de todos os pacientes analisados apresentarem OAJ grave. Welhaven et al. 22 realizaram análise do líquido sinovial de pacientes com lesão meniscal e/ou do ligamento cruzado anterior, concluindo que homens e mulheres possuíam perfis metabólitos distintos, e sugeriram que esses dados poderiam ser usados no futuro para avaliação do desenvolvimento de OA pós-traumática. Por outro lado, Zhai et al. 23 sugeriram que a fenilalanina poderia ser um marcador plasmático para a progressão da OAJ radiográfica bilateral em mulheres. No presente estudo, as análises foram realizadas apenas no líquido sinovial de pacientes com OAJ primária, o que poderia justificar a diferença entre os achados.

A análise multivariada não demonstrou diferenças no metabolismo do líquido sinovial de acordo com o IMC. Este resultado nos surpreendeu, pois já foi demonstrada a existência de diferenças significativas no metabolismo sérico entre obesos e não obesos com OAJ, porém analisando amostras de sangue. 24 Por se tratar de uma doença sistêmica, é possível que o perfil de metabólitos no sangue de obesos e não obesos não seja equivalente ao do ambiente sinovial. Outra explicação plausível para o nosso resultado é a influência da subclassificação que utilizamos, estratificada em três grupos, em vez da comparação entre obesos e não obesos. Ainda que a análise multivariada não tenha encontrado diferença, a concentração sinovial da glicose foi inferior no grupo de obesos. Embora o metabólito glicose não seja apontado diretamente como um biomarcador relacionado ao IMC, sabe-se que a elevação de metabólitos como a manose vincula-se com o sobrepeso e com a obesidade. 25 Especulamos que na articulação dos indivíduos obesos ocorra um maior consumo de glicose pelas células sinoviais.

As comparações do perfil metabólico do líquido sinovial entre pacientes com e sem diabetes e de acordo com o valor sérico da glicemia de jejum não evidenciaram diferenças nas análises multivariadas e univariadas. A relação entre diabetes e OA vem sendo muito pesquisada e já foi sugerida a existência de um fenótipo induzido pelo diabetes. 26 Um fator que pode ter influenciado nosso resultado é que não foi considerado o uso de hipoglicemiantes ou de insulina, que interferem diretamente no metabolismo da glicose. Além disso, a classificação entre diabéticos e não diabéticos foi determinada por autodeclaração na entrevista e não por métodos de diagnóstico laboratorial.

A hipertensão parece contribuir na patogênese da OA por aspectos bioquímicos e biomecânicos, ao interferir na microcirculação articular. 27 A análise multivariada não foi capaz de distinguir os grupos de pacientes com ou sem hipertensão de acordo com o perfil metabólico. Entretanto, os metabólitos valina, arginina e citrato apresentaram níveis mais elevados nos indivíduos sem a doença. Este resultado sugere um consumo de citrato em favor das vias inflamatórias na articulação destes indivíduos, corroborando com a hipótese de maior influência inflamatória na OA associada a essa doença crônica. 27 A valina também demonstrou menor concentração sinovial nos hipertensos. A elevação da razão sérica leucina/histidina também pode servir como biomarcador para o diagnóstico de OAJ estando este aumento relacionado à degradação do colágeno. 19 A arginina foi o outro metabólito que expressou redução no grupo com hipertensão. Zhang et al. 28 apontaram a redução sérica da arginina como um possível biomarcador de osteoartrite avançada, inferindo que a redução da arginina sérica se origina de um aumento compensatório na síntese de colágeno na OA grave. Outra pesquisa demonstrou o aumento dos níveis séricos de arginina em pacientes com OA grave, submetidos à ATJ e do quadril, comparado com pessoas saudáveis. 29 A redução na concentração sinovial de alguns metabólitos nos pacientes com OA e hipertensão poderia ser justificada por uma perturbação na drenagem sinovial, decorrente do comprometimento vascular nesta condição. 27 Por esta hipótese, a manutenção destes substratos por mais tempo no ambiente articular elevaria as suas taxas de metabolização aumentando os níveis dos seus subprodutos. Werdyani et al. 30 evidenciaram após análise metabolômica do plasma de pacientes com OA de quadril e joelho três endotipos da doença: fraqueza muscular, déficit de arginina e inflamação de baixo grau. Embora tenham analisado o plasma, tais achados corroboram nossos dados, uma vez que os pacientes hipertensos apresentavam menores níveis de arginina.

Quanto à avaliação do perfil metabólico do líquido sinovial nos pacientes classificados como com ou sem doença cardíaca, não encontramos diferenças nas análises. Contudo, o diagnóstico de doença cardíaca foi baseado na avaliação pré-anestésica, e apenas 6% da amostra foi classificada como portadora deste grupo de doenças.

Um dos pontos fortes do nosso estudo foi o número de amostras de líquido sinovial de pacientes com OAJ. Outro ponto positivo da pesquisa foi a menor variabilidade em relação ao grau de doença articular, comparando pacientes apenas com estágios avançados de destruição articular.

Uma das principais limitações do trabalho foi a classificação de algumas condições clínicas baseada em dados da ficha padrão de avaliação pré-anestésica, por autodeclaração. Outra limitação foi a realização da análise apenas do líquido sinovial, limitando a comparação com outros estudos que utilizaram o plasma.

## Conclusão

As análises multivariadas do líquido sinovial não evidenciaram diferenças entre os pacientes com OAJ de acordo com as características clínicas. Os metabólitos 3-hidroxibutirato e L-valina apresentaram níveis menores em função da gravidade radiográfica, os pacientes obesos apresentaram menor concentração de glicose, enquanto os pacientes hipertensos apresentaram menores valores de arginina, valina e citrato.
